# Beyond brain injury biomarkers: chemoattractants and circulating progenitor cells as biomarkers of endogenous rehabilitation effort in preterm neonates with encephalopathy

**DOI:** 10.3389/fped.2023.1151787

**Published:** 2023-05-24

**Authors:** N. Efstathiou, V. Soubasi, G. Koliakos, K. Kantziou, G. Kyriazis, A. Slavakis, V. Dermentzoglou, I. Michalettou, V. Drosou-Agakidou

**Affiliations:** ^1^ 1st Neonatal Department and NICU, Hippokration General Hospital, Aristotle University of Thessaloniki, Thessaloniki, Greece; ^2^ 2nd Neonatal Department and NICU, Papageorgiou General Hospital, Aristotle University of Thessaloniki, Thessaloniki, Greece; ^3^ Biochemistry Department, Medical School, Aristotle University of Thessaloniki, Thessaloniki, Greece; ^4^ Immunology Laboratory, Pulmonology Department, Papanikolaou General Hospital, Aristotle University of Thessaloniki, Thessaloniki, Greece; ^5^ Biochemistry Department, Hippokration General Hospital, Thessaloniki, Greece; ^6^ Child Radiologist, Radiology Department, Agia Sofia Pediatric Hospital, Athens, Greece; ^7^ Child Occupational Τherapist, Hippokration General Hospital, Thessaloniki, Greece

**Keywords:** neonate, preterm, encephalopathy, biomarkers, S100B, NSE—neuron-specific enolase, progenitor cells, stem cells

## Abstract

**Introduction:**

Preclinical work and studies in adults have shown that endogenous regeneration efforts that involve mobilization of progenitor cells take place after brain injury. However, kinetics of endogenous circulating progenitor cells (CPCs) in preterm neonates is not well described, particularly their possible role regarding brain injury and regeneration. We aimed to assess the kinetics of CPCs in neonates with encephalopathy of prematurity in relation to brain injury biomarkers, chemoattractants and relevant antenatal and postanal clinical factors, in an effort to outline the related pathophysiology.

**Materials and methods:**

47 preterm neonates (of 28–33 weeks GA) were enrolled: 31 newborns with no or minimal brain injury (grade I IVH) and 16 prematures with encephalopathy (grade III or IV IVH, PVL or infarct). Peripheral blood samples obtained on days 1, 3, 9, 18 and 45 after birth were analyzed using flow cytometry, focusing on EPCs (early and late Endothelial Progenitor Cells), HSCs (Hematopoietic Stem Cells) and VSELs (Very Small Embryonic-Like Stem Cells). At the same time-points serum levels of S100B, Neuron-specific Enolase (NSE), Erythropoietin (EPO), Insulin-like growth factor-1 (IGF-1) and SDF-1 were also measured. Neonates were assessed postnatally with brain MRI, and with Bayley III developmental test at 2 years of corrected age.

**Results:**

Preterms with brain injury proved to have significant increase of S100B and NSE, followed by increase of EPO and enhanced mobilization mainly of HSCs, eEPCs and lEPCs. IGF-1 was rather decreased in this group of neonates. IGF-1 and most CPCs were intense decreased in cases of antenatal or postnatal inflammation. S100B and NSE correlated with neuroimaging and language scale in Bayley III test, providing good prognostic ability.

**Conclusion:**

The observed pattern of CPCs’ mobilization and its association with neurotrophic factors following preterm brain injury indicate the existence of an endogenous brain regeneration process. Kinetics of different biomarkers and associations with clinical factors contribute to the understanding of the related pathophysiology and might help to early discriminate neonates with adverse outcome. Timely appropriate enhancement of the endogenous regeneration effort, when it is suppressed and insufficient, using neurotrophic factors and exogenous progenitor cells might be a powerful therapeutic strategy in the future to restore brain damage and improve the neurodevelopmental outcome in premature infants with brain injury.

## 1. Introduction

Premature birth remains a leading cause of childhood mortality and morbidity worldwide (1). Advances in therapeutic protocols in neonatal intensive care unit (NICU) led to increased survival of even more immature infants, increasing the susceptibility of the immature brain to damage and the clinical challenge for neuroprotection (2). Currently, no specific therapeutic strategies are applied in routine clinical care to actively repair brain injury. The principal forms of encephalopathy in prematures are periventricular leukomalacia (PVL) and intraventricular hemorrhage (IVH) (3, 4). PVL is a white matter injury defined as a focal periventricular necrosis and a more diffuse reactive gliosis and microglial activation (5). Ischemia (mainly) and infection/inflammation lead to degeneration of late oligodendrocyte progenitors (preOLs) in the acute phase (3). It seems that early oligodendrocyte progenitors are capable of regenerating preOLs, but the latter fail to maturate and progress to normal myelination at the end (6). IVH is the most common variety of intracranial hemorrhage in preterms, starting from the subependymal germinal matrix and expanding till the periventricular space (3). The main pathogenetic mechanisms involve intravascular, vascular and extravascular factors that are affected from multiple other parameters of a critical ill preterm in the NICU (7).

*Β*rain injury is often biochemically defined in preclinical and clinical studies with the use of brain biomarkers (8). Among them, S100B and Neuron Specific Enolase (NSE) have proved to be more hopeful (8). **S100B**, a calcium-binding protein that is produced primary in astrocytes, is detectable in cerebrospinal fluid (CSF), in blood circulation and in urine after brain injury (9, 10). Likewise, **NSE**, a glucose metabolism isoenzyme, that is produced mainly in neurons and neuroendocrine cells, is detectable in CSF and blood after brain damage (10).

A plethora of neuroprotective strategies have been studied in neonatal brain injury, but most of them did not obtain results that can be used in a clinical setting (9). Lately, the possible therapeutic role of neurotrophic factors and progenitor cells have won a great part of scientific interest. **Erythropoietin (EPO)** is a 30.4 kDa endogenous glycoprotein with known pleiotropic properties (11). In addition to its role in erythropoiesis, EPO is known for its neuroprotective role after hypoxia (12). Specifically, a few hours after a hypoxic-ischemic event, the expression of EPO and its receptor is increased in neurons and endothelial cells, and after days in astrocytes (13–15). Its beneficial mechanisms of action include antiexcitotoxic, antioxidant, antiapoptotic effects, as well as promotion of angiogenesis and neurogenesis (12). Caspase-3 inhibition and resultant antiapoptotic actions and thus cell survival begins with adaption of EPO to its receptor in cell surfaces (6). Most types of neural cells express EPO receptor (11). It seems that after brain injury EPO receptors are upregulated, but EPO ligand increase is insufficient and thus cell apoptosis is not prevented (11). But EPO has also neurorestorative properties in the subacute phase, promoting differentiation of pre-OL, and thus normal myelination (6). Finally, EPO act as a chemotactic factor and mobilizes endogenous progenitor cells (16). In the central nervous system, EPO is basically secreted by astrocytes (11). Multiple studies in animals (16–18) and in neonates (6, 11, 17, 19–24) obtained positive results, mostly histological and less functional, but inconsistency among researchers is remarkable and thus clinical usefulness is hampered (6). Dose, dosage timing and repeated doses are critical parameters that influence the outcome and there is a need to be further studied. **Insulin-like Growth Factor-1 (IGF-1)** is a growth factor that is essential for maturation of the fetal brain and differentiation of pre-oligodendrocytes. It has antiapoptotic actions and enhances survival of neural cells (25). It seems that its effect is beneficial in low doses and cytotoxic in higher ones (7).

Progenitor cells are multipotent cells of primitive origin that take part in the process of organogenesis intrauterine, and in tissue repair in the extrauterine life. They have the ability of self-renewal and differentiation into multiple cell lines (plasticity) when appropriate growth factors exist (26, 27). They are found in blood (**circulating progenitor cells- CPCs**), in tissues and in the bone marrow, and they are mobilized in a chemotactic way to the target-organ via chemoattractants which are released from the latter (26, 28–34). Their main mechanisms of action are anti-inflammatory and trophic action, neoangiogenesis and neurogenesis (35–37). Preclinical models (38–40) and data from clinical studies (27, 41–42) have shown that progenitor cells derived both topically, from the opposite brain hemisphere and from periphery can be mobilized toward the place of brain injury, especially considering that the blood-brain barrier is more permeable in cases of brain damage. The most promising progenitor cell lines studied are the Hematopoietic Stem Cells (HSCs), the Very Small Embryonic-like Stem Cells (VSELs) and the Endothelial progenitor cells (EPCs). **HSCs** are mainly found in the bone marrow. They take part in hematopoiesis (43), but also in regeneration after tissue injury (44) via the chemoattractant SDF-1 and its receptor CD184 (45–48). Preclinical (49) and clinical studies in adults (50–52) after ischemic stroke have shown CD34+ cells to be mobilized and correlate with improved outcome. **Stromal cell-derived factor-1 (SDF-1)** is a chemoattractant factor for cells that have CD184 (CXCR4) receptor in their surface. It guides circulating cells to the injured tissue, or homing back to the bone marrow (53, 54). In uterus, SDF-1 has a basic role in guiding neuroblasts and in organogenesis (54), procedures that are abruptly interrupted after premature delivery. **VSELs** are cells of non-haemopoietic origin, that are lately found mainly in the cord blood and bone marrow, and take part in organogenesis and tissue regeneration (55). They are mobilized via SDF-1/CD184 axis and Hepatocyte Growth Factor (55, 56). Preclinical (56) and clinical studies in adults (53, 57) have shown mobilization and regeneration effort after stroke. **EPCs** are mainly found in bone marrow and migrate via cytokines [as erythropoietin and vascular endothelial growth factor (VEGF)] to the periphery for angiogenesis and neoangiogenesis purposes (58–60). Late (lEPCs) and early (eEPCs) endothelial progenitor cells are the two main cell lines lately described. Studies in animals (61) and adults (62–65) have shown that EPCs are increased in acute tissue injuries and decreased in chronic vascular diseases. Low oxygen enhances EPO, IGF-1 and SDF-1 release via Hypoxia-inducible factor 1-alpha (HIF-1a) (54, 66). Methodological differences in the design of these studies make comparisons difficult and impede the extraction of certain conclusions. Histochemical improvement is usually observed, but clinical long-term outcome is mostly disappointing.

This great inconsistency in the results of both preclinical models and clinical trials has hampered their clinical usefulness so far. Even more, studying limited parameters in each study and at limited time-points provides a restricted knowledge and not the whole picture, the whole biochemical frame of pathophysiological events. Awaiting of new trials with different dosages and timing of administration would provide more useful data and a possible clinical utility in the future. In this study, we speculated another way for extracting useful clinical data. We assumed that for substances and cell lines that are endogenously present in the neonate, studying the related pathophysiology (i.e., how the human body is using these molecules and cells after a deleterious event) would provide more clues for their clinical importance. To be more specific, we hypothesized that if endogenous progenitor cells are not mobilized at all after neonatal brain injury (i.e., if the human body -in its relative perfection after centuries of evolution- does not use them), it would be rather impossible that exogenous administration of them be beneficial. Similarly, if EPO and IGF-1 levels in serum are not changed after brain injury, it would be rather impossible that systematically administration of them to be of value. Evolution through centuries has probably done thousands of “trials” itself, promoting only the biochemical ways that enhance survival. We believe that mimicking biochemical and cellular patterns that promote recovery (as it is shown in cases of favorable outcome) or enhancing pathways that are malfunctioning (in cases of adverse outcome) is of great clinical value.

**Figure 1:**
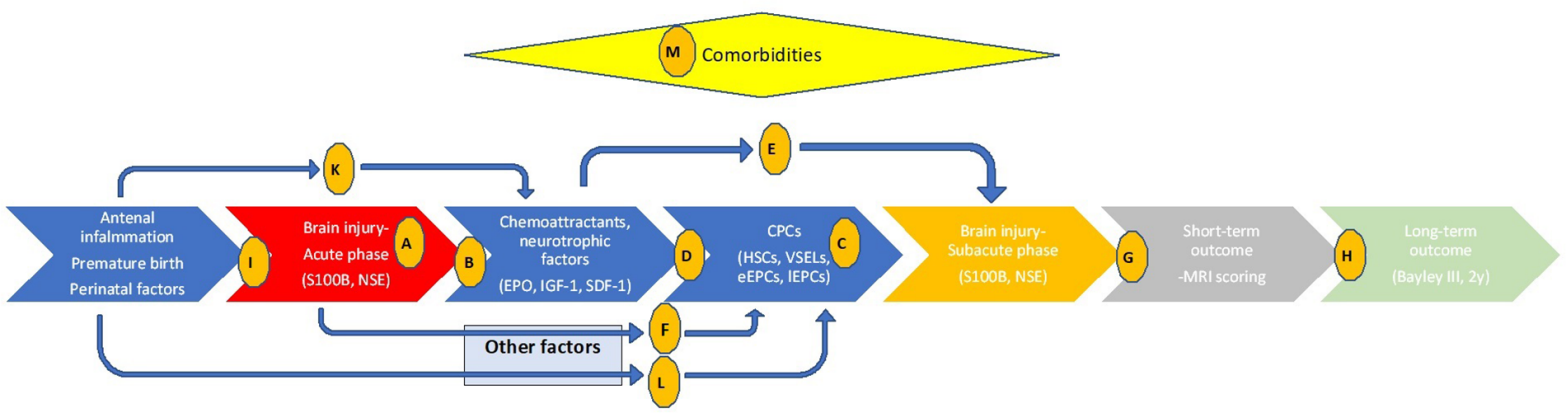
Timeline of events related to preterm brain injury, relations between clinical and biochemical parameters, and hypothesis investigated in the current study. Letters in circles indicate the hypothesis studied. Hypothesis A-M: (**A**) the timeframe of brain injury (S100B and NSE). (**B**) kinetics of neurotrophic and chemotactic factors (EPO, IGF-1 and SDF-1). (**C**) kinetics of CPCs (HSCs, VSELs, eEPCs, lEPCs). (**D**) Do neurotrophic factors/chemoattractants of interest correlate with CPCs? (**E**) Do neurotrophic factors of interest have neuroprotective role themselves? (**F**) Does brain injury alters CPCs mobilization via other biochemical factors? (**G**) Is there a correlation of parameters studied to MRI scoring? (**H**) Is there a correlation of parameters studied to Bayley III scoring? (**I**) Do antenatal clinical factors relate to the brain injury themselves? (**K**) Do antenatal clinical factors affect neurotrophic factors/chemoattractants? (**L**) Do antenatal clinical factors affect CPCs? (**M**) Do comorbidities affect parameters studied?

In this single-center pilot translational study, we intend to illustrate related pathophysiology and endogenous regeneration biochemical pathways directly in human preterm neonates with encephalopathy, aiming to confirm if preclinical observations are valid in real clinical situations. The primary aim is to explore the timeframe of brain injury using brain injury biomarkers (S100B and NSE) as a baseline for the biochemical correlations (hypothesis A, Figure 1) in preterms with brain injury, in preterms without obvious brain injury and in healthy full-term neonates. Similarly, we intend to investigate kinetics of possible neurotrophic and chemotactic factors (EPO, IGF-1 and SDF-1) (hypothesis B), and kinetics of some of the most promising progenitor cell lines (HSCs, VSELs, eEPCs, lEPCs) (hypothesis C) in these infants. Secondary aims are to explore if the neurotrophic factors we chose to investigate act as chemoattractants for CPCs in the current study (hypothesis D), or if they have neuroprotective role themselves (independently of CPCs' mobilization) (hypothesis E). Additionally, we aim to investigate if brain injury alters CPCs' mobilization via other biochemical factors that are not studied here (hypothesis F) and mostly if there is a correlation of all these parameters to neuroimaging (hypothesis G) and long-term neurodevelopmental outcome (hypothesis H). Finally, we explore if prenatal clinical factors -that are known to implicate premature birth and its complications- are related to the extent of brain injury themselves (hypothesis I), or affect neurotrophic factors/chemoattractants (hypothesis K) or progenitor cells (hypothesis L) mediating to the neurorestoration process. Interference of comorbidities are also investigated (hypothesis M, Figure 1). Even knowing that biochemical pathways are very complicated, even more in the critical setting of a premature in the NICU with multiple comorbidities and prenatal etiologies, in this study we provide a translational starting point that includes all these parameters simultaneously in multiple time-points, with the additional neuroimaging, short- and long-term outcome as the final point of possible clinical utility.

## 2. Materials and methods

### 2.1. Patients

Preterm newborns with a gestational age of less than 33 weeks who were born or admitted to our tertiary neonatal intensive care unit within the first 24 h of life were enrolled and studied prospectively from the first day of life. Newborns that developed IVH ≥ II grade, PVL or infarct were compared to prematures without obvious or minimal (IVH I grade) brain damage, who were considered as preterm controls. IVH was diagnosed based on serial cranial ultrasounds. PVL and infarct were diagnosed using both cranial ultrasounds and magnetic resonance imaging techniques (MRI). A group of healthy full-term neonates also participated for age-related correlations between newborns without brain injury. Factors that could influence biomarkers levels were considered as reasons for *exclusion* from the study, namely intrauterine growth retardation, major congenital or metabolic disorders, and twins with death or major abnormalities in their siblings (67–70). Hemolyzed samples were also excluded. Data on demographic, perinatal characteristics, and prematurity-associated complications were collected prospectively for all patients. The study was approved by the Ethical Committee of the Faculty of Medicine, Aristotle University of Thessaloniki, and the Scientific Committee of Hippokration Hospital. Written consent was obtained from the parents of the newborns.

Peripheral blood samples were obtained on days (d) 1 (12–24h of life), 3, 9, 18 and 45 of life and analyzed using flow cytometry, focusing on HSCs, VSELs, eEPCs and lEPCs. From the same blood samples serum and plasma was collected and stored at −80*^ο^* C until measurement of biochemical factors (brain injury biomarkers and chemoattractants).

### 2.2. Flow cytometry

Blood samples were incubated with monoclonal antibodies [PEantiCD184/CXCR4, FITCH-antiCD34, PECy5-antiCD45 (BD Biosciences, San Jose, CA, USA)] and APC-antiCD133 (Miltenyi Biotec, Bergisch Gladbach, Germany) and analyzed with a BD FACSCalibur instrument (BD FACSDiva Software, version 6, BD Biosciences, San Jose, CA, USA). 100,000 events were acquired. Levels of each cell population were expressed as a percentage of the total events. CPCs of interest were populations enriched in HSCs (CD34^+^/CD184^+^/CD45^+^), VSELs (CD34^+^/CD184^+^/CD45^−^), eEPCs (CD34^+^/CD45^dim/−^/CD184^+^/CD133^+^) and lECPs (CD34^+^/CD45^dim/−^/CD184^+^/CD133^−^). A detailed description of flow cytometry methodology and gating strategy has been described earlier (27).

### 2.3. Biochemical assessment

S100B and NSE were assessed using Immunochemiluminometric assay (ICMA) (Liaison, DiaSorin-SpA, Saluggia, Italy) according to the manufacturer's instructions. Serum levels of EPO and IGF-1 were measured using immunochemiluminometric assay (Immulite 2000XPi, Siemens, Llanberis, United Kingdom), and plasma levels of SDF-1 were assessed using ELISA (Quantikine ELISA, R & D Systems, Minneapolis, USA).

### 2.4. Neurodevelopmental assessment

Neurodevelopmental assessment was performed after the second year of corrected age by an experienced investigator who was unaware of the patients' medical history. Baley III test and its scales were used to examine cognitive, language (receptive and expressive) and motor (fine and gross) domains (71). Neurodevelopmental impairment (NDI) was defined as *mild* when Bayley scores were between −1 and −2 standard deviations (SD) of the mean provided by the test, and as *moderate/severe* when scores were lower than −2 SDs (72).

Brain MRI was performed in preterms with encephalopathy at term-equivalent postmenstrual age. MRI abnormalities in white matter (cystic degeneration, focal signal abnormalities, delayed myelination, thinning of the corpus callosum, dilated lateral ventricles, reduction of white matter volume), in cortical grey matter (signal abnormality, delayed gyration, dilated extracerebral CSF space), in deep grey matter and in cerebellum, as well as a total MRI brain injury score, were evaluated as Kidokoro et al. have proposed (73).

### 2.5. Statistical analysis

Data were presented as mean (±SD) or median (range) depending on distribution, and t-test or the Mann-Whitney U-test was used for quantitative parameters respectively. Likewise, Pearson's correlation or Spearman's rank correlation were used for correlations accordingly to distribution, and Pearson Chi-Square or Fisher's exact test were used for qualitative variables. Receiver operating characteristics (ROC) curves were assessed using the areas under the curves as indicated. Sample size analysis was based in previous exploratory study (27) and was used to indicate the lowest sample size required to detect statistical significance. Alpha was set at 0.05% and 80% power was accepted. Calculating for an additional 10% drop-out rate, estimated sample size was defined as 11 neonates per arm. SPSS statistical package was used (SPSS statistics, IBM Corporation, version 20, Chicago, IL, USA). *P* value of less than 0.05 was considered as statistically significant. As this is a pilot study, when rational causalities exist, borderline *p* values (0.05 > *p* > 0.08) were also reported to indicate trends.

## 3. Results

### 3.1. Neonatal characteristics

47 preterm neonates were included in the study and were divided into two groups based on neuroimaging findings: patients (group 1, *n* = 16) included neonates with IVH of >II grade, PVL or infarct, whereas preterm controls (group 2, *n* = 31) included prematures with no (*n* = 28) or minimal (IVH/I grade, *n* = 3) findings of brain injury. An additional group of healthy full-term newborns was studied for comparison reasons (group 3, *n* = 11). Neonatal characteristics are presented on Table 1.

**Table 1:** Demographic and perinatal characteristics of the three group of neonates studied.

Groups	1 Preterm Patients	2 Preterm Controls	3 Full-term Controls	Comparisons (*p*)
*n*	16	31	11	
Chorioamnionitis [*n* (%)]	3 (18.8%)	4 (12.9%)	0	-
Antenatal Steroids [*n* (%)]	7 (46.6%)	30 (96.8%)		1–2 (<0.0005)
PRoM [*n* (%)]	3 (20%)	9 (29%)	0	-
Caesarean section [*n* (%)]	9 (56%)	23 (74%)	6 (54%)	-
Multiple gestation [*n* (%)]	4 (26.6%)	15 (48.3%)	2 (18%)	-
Gestational Age (weeks) [Mean (SD)]	27.3 (2.9)	29.8 (2.1)	38 (1.2)	1–2 (<0.005)
				2–3 (<0.0005)
Birth Weight (gr) [Mean (SD)]	1,182 (495)	1,526 (396)	2,988 (330)	1–2 (<0.01)
				2–3 (<0.0005)
Male gender [*n* (%)]	7 (43.8%)	16 (51.6%)	5 (45%)	-
Apgar Score 1′ [median(range)]	3 (7)	8 (7)	8 (1)	1–2 (<0.0005)
Apgar Score 5′ [median(range)]	5 (9)	9 (5)	9 (1)	1–2 (<0.0005)
SBE (first hours of life, mmol/L) [Mean (SD) ]	−12.30 (8.65)	−5.46 (3.52)	−6.79 (3.1)	1–2 (<0.01)
IVH (I/) [*n* (%)]	0	3 (9.6%)	0	-
IVH (II) [*n* (%)]	2 (12.5%)	0		
IVH (III/IV) [*n* (%)]	10 (62.5%)	0	0	
PVL [*n* (%)]	6 (37.5%)	0	0	
Infarct [*n* (%)]	1 (6.2%)	0	0	
RDS [*n* (%)]	7 (46.7%)	15 (48.4%)	2 (16.7%)	-
BPD [*n* (%)]	5 (33.3%)	5 (16.1%)		-
NEC [*n* (%)]	2 (12.5%)	9 (29%)	0	-
ROP (>3rd grade) [*n* (%)]	2 (13.3%)	1 (3.2%)	0	-
Sepsis (late) [*n* (%)]	2 (12.5%)	2 (6.5%)	0	-
Hospitalization duration (in days) (Mean/SD)	66.5 (29.6)	45.1 (25.6)		-
Death [*n*] [day of death: median (range)]	9 11 (53)	2 31.5 (51)	0	1–2 (<0.0005)

RDS, respiratory distress syndrome; BPD, bronchopulmonary dysplasia; IVH, Intraventricular hemorrhage; NEC, necrotizing enterocolitis; PRoM, premature rupture of membranes (>24 h); PVL, periventricular leukomalacia; ROP, retinopathy of prematurity; SBE, standard base excess.

### 3.2. S100B

#### 3.2.1. Kinetics

**Figure 2:**
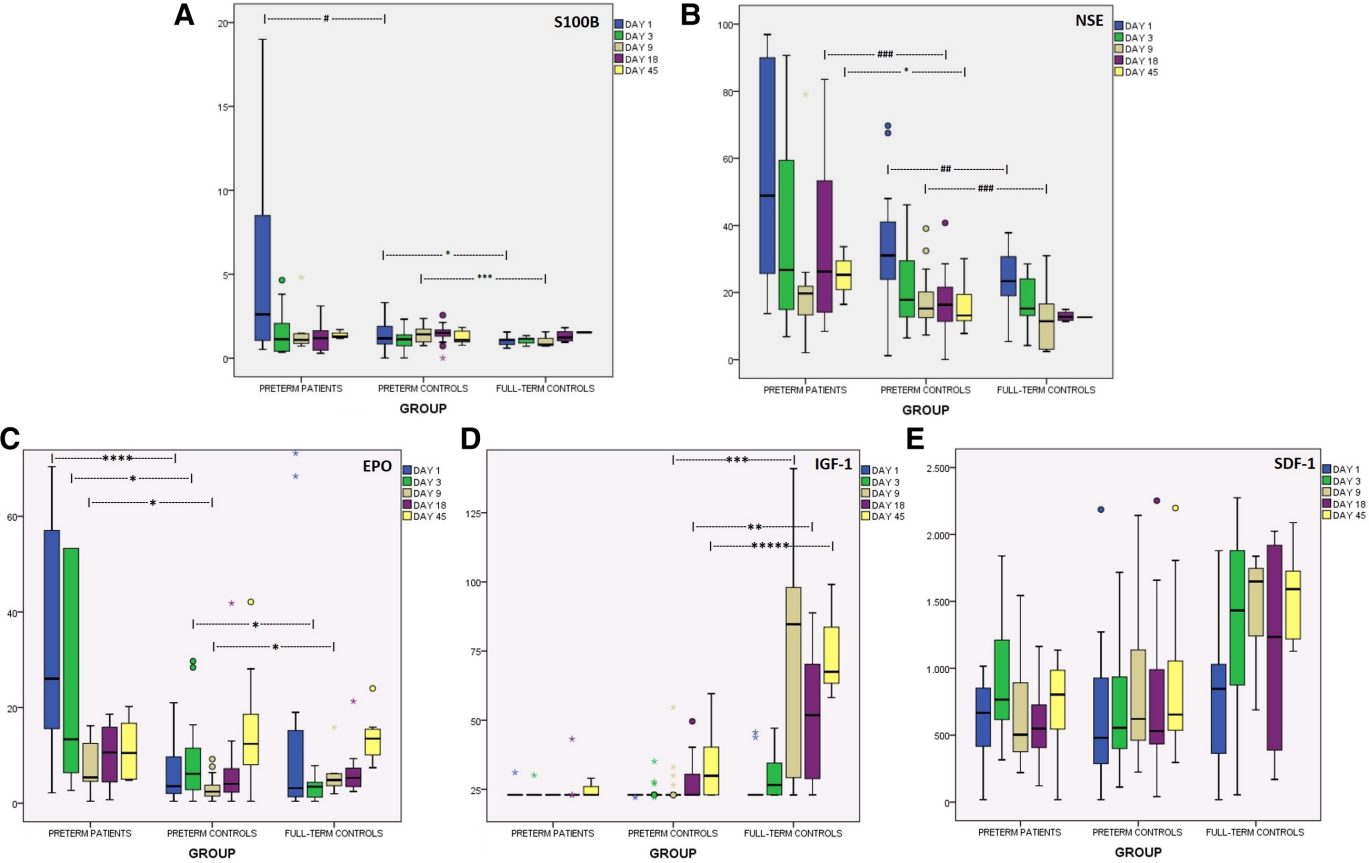
Kinetics of brain injury markers and chemoattractants in the first 45 days of life, in preterms with encephalopathy, in preterm controls and in full-term controls. (**A**) S100B (μg/L), (**B**) NSE (μg/L), (**C**) EPO (mIU/ml), (**D**) IGF-1 (ng/ml), (**E**) SDF-1 (pg/ml). *p < 0.05, **p < 0.01, ***p < 0.005, ****p < 0.001, *****p < 0.0005. ^#^*p* = 0.06, ^##^*p* = 0.068, ^###^*p* = 0.08.

Preterms with encephalopathy presented higher S100B levels than preterm controls on day 1, mainly the ones with severe IVH (grade III/IV). A cut-off point of 2.51 μg/L on that day provided good prognostic ability with regards to the development of severe IVH (Table 2). Infants with PVL had borderline increased S100B levels on day 18. Moreover, S100B was increased in the preterm controls compared to the full-term controls on days 1 and 9. Kinetics of S100B are presented in Figure 2 and Table 2.

**Table 2:** Results of brain injury markers, chemoattractants and CPCs in preterms with encephalopathy (when compared to preterms controls).

Biomarker	Prenatal/Perinatal Factors	d1	d3	d9	IVH (grade)	d18	d45	Comorbidities	MRI	2y-Bayley	Sens %	Spec %	AUC	r/ rho	p	Hypothesis tested
**S100B** (μg/l)		**7.3[8.4]** *^ac^ **(vs 1.4[0.8])**	1.7[1.5] *^a^ (vs 1.1(0.6]	1.5[1.3] *^a^ (vs 1.4[0.4]		1.3[0.9] *^a^ (vs 1.5[0.5]	1.4[0.3] *^a^ (vs 1.2[0.4]								d1:<0.005 d3-D45:ns	A
		**↑** **2.51**			III/IV						71.4	90.3		0.756	<0.05 <0.05	G
						↑			PVL						0.08	G
		**2.19** **1.32**								RS<1SD	60 80	94/64.7	0.835		<0.05	H
	CS			↑											0.06	I
	↓ApSc1'	**↑**												-0.33	<0.05	I
	SBE	**↑**												-0.42	<0.01	I
		**↑**						seizures							<0.005	M
		**↑**						Late sepsis							<0.05	M
		↑^c^						death							0.071	H
		↑						RDS III/IV							0.069	M
**NSE** (μg/l)		72.8 [67.5] (vs 34.9[19]) *^a^	38.1 [30] (vs 25.5[26]) *^a^	23.3 [30.1] (vs 20.9[21]) *^a^		35.9 [26] (vs 16.6[9]) *^a^	**25.1 [8.6]** (vs **15[5.7**]) *^a^								d18:0.078 d45:<0.05 d1/3/9:ns	A
			**↑**		III/IV	**↑**	**↑**								<0.05 <0.01	G
			** **			** **	**22.6**		>8^i^		100	93.3	0.967		<0.05	G
		**57.8**	** **				** **			LS<1SD RS<1SD	75 80	88.9 100	0.889 0.953		<0.05	H
			** **	↑				RDS III/IV							0.07	M
**EPO** (mIU/ml)	** **	**14.8 [24.7]** (vs 3.6[21]) *^b^	**13.4[12]** (vs 6.6[29.3] *^b^	**4.7[6.6]** (vs 2.4[8.8]) *^b^		3.5[13.7] (vs 4[41.4]) *^b^	10.3[15.4] (vs 11.2[28]) *^b^								d1: <0.001 d3/9: <0.05 d18/45:ns	B
		** **	**↑** ^c^	**↑** ^c^	III/IV		** **								<0.05	G
		↑^c^	** **	** **			** **	PVL							0.059	G
	MultPreg	↓		** **			** **								0.052	K
	PreSter	↑		** **			** **								0.08	K
	↓ApSc1/5'	**↑**		** **			** **							-0.55	<0.001	K
	ApSc1'(<4)	** **	↑	** **			** **								0.064	K
	↓pH	**↑**	**↑**	** **			** **							-0.5	<0.05	K
			↑S100B^c^	S100B<>↑^c^		**↑**								0.67 0.47	0.07 <0.05	B
		↑ ^c^ ↑ ^c^	**↓S100B**	↓S100B										-0.5 -0.39	<0.05 0.075	E
			↑NSE <>↑^c^	** **		** **	** **							0.43	<0.05	B
		↑<>**lEPCs**^d^		** **		** **	** **							0.59	<0.05	D
		↑	↑			↑eEPCs^d^ **↑eEPCs**^d^								0.77 0.75	0.07 <0.05	D
			↑			**↑HSCs** ^d^								0.42	<0.05	D
		**↑**	**↑**					RDS (III/IV)							0.07 <0.05	M
**IGF-1**	ApSc1'(<4)					**↓**	**↓**								<0.05	K
					III/IV	↓									0.08	G
		↑S100B^c^ ↑S100B				**↓** ^c^	**↓**							-0.35 -0.51	0.077 <0.05	B
		↑NSE ↑NSE * *	↑NSE * *	↑NSE		**↓** ↑NSE<>**↓**	**↓** ↓ **↓**							-0.36 -0.68 -0.37 -0.44 -0.39	<0.05 <0.0005 0.072 <0.05 <0.05	B
				**↓** ** **		↓	**↓**	Late sepsis							<0.05 0.08	M
						**↓**		RDS (III/IV)							<0.05	M
						↓	**↓**	BPD							0.079 <0.05	M
						**↓** ^c^	**↓**	durHosp						-0.45	<0.05	M
			**↓**			↓		death							<0.05 0.08	H
**SDF-1**			↓		III/IV III/IV		↓								<0.05 0.07	G
		↓						PVL							0.059	G
	PreSter	↓													0.055	K
	Chorioam	↓													0.08	K
		↑S100B		**↓** ↑S100B<>↑ ↑S100B		**↑** ↑S100B<>**↑**	↑S100B<>↑							-0.41 0.33 0.46 0.52 0.5	<0.05 0.08 <0.05 <0.05 0.08	B
		↑NSE		**↓** ↑NSE		↑								-0.45 0.36	<0.01 0.067	B
			↑<>**↑**VSELs											0.42	<0.05	D
				↑		↑HSCs ↑<>**↑**HSCs								0.32 0.39	0.08 <0.05	D
						↑<>**↑**eEPCs								0.39	<0.05	D
		**↑**						**RDS (III/IV)**							<0.05	M
							**↓**	**BPD**							0.077	M
**HSCs**			↑* ^g,h^	↑*^g,h^											<0.05 <0.05	C
	MultPreg	**↓**	**↓**												<0.0005 <0.05	L
	Chorioam	**↓**		↓		↓									<0.05 0.08 0.06	L
	↓ApSc1' ApSc1’ <4		**↑** ** **	↑										-0.38	<0.05 0.07	L
		↑S100B ↑S100B	**↓**	↓										-0.35 -0.31	<0.05 0.07	F
			↑NSE			↑ ↑NSE>>**↑**								0.33 0.54	0.068 <0.005	F
		**↓** **<0.017‰**^e^	**↓**	**↓**		**↓**	**↓**	Late sepsis			100	66.7	0.811		<0.05 0.071 <0.005 <0.05 <0.01 <0.05	M
			**↓** **<0.015‰**^e^					NEC			66.7	66.7	0.739		<0.05 <0.05	M
**VSELs**				↑				**PVL**							0.056	G
	Chorioam		**↓**												<0.05	L
	ApSc1' (<4)	↓				**↓**	↓								0.055 <0.05	L
		S100B<>**↓^c,g^** ↑S100B**^c,g^** ↑S100B**^c,g^**	↓				**↓**							-0.49 -0.35 -0.67	<0.01 0.078 <0.005	F
			↑NSE			**↑** ↑NSE>>**↑**								0.44 0.42	<0.05 <0.05	F
		**↓** **<0.014‰** ^e^	↓	**↓** ** **		**↓**	**↓**	Late sepsis			100	79.5	0.897		<0.01 <0.01 0.08 <0.0005 <0.05	M
						**↓**		RDS (III/IV)							<0.05	M
						**↓**		BPD							<0.05	M
**eEPCs**			↑*	↑*											<0.005 <0.0005	C
			**↑**	**↑**	III/IV										<0.01 <0.005	G
						↑		PVL							0.08	G
			↑NSE>>↑			↑NSE>>↑								0.33 0.6	0.065 <0.001	F
	MultPreg	**↓**	↓	**↓**											<0.005 0.06 <0.05	L
	↓ApSc1/5'		**↑**	**↑**										-0.48	<0.05	L
							↑	durHosp						0.36	0.08	M
				↑				death							<0.05	H
		↓	↓			**↓**		Late sepsis							0.075 <0.05	M
			↓ **<0.0095‰**^e^					NEC			66.7	70	0.724		<0.05 <0.05	M
**lEPCs**		↑*	↑*	↑*											<0.05 0.056	C
		↑S100B ↑S100B	↓			**↓** ^g^								-0.31 -0.37	0.075 0.059	F
			↑NSE			**↑** ↑NSE<> **↑** ↑NSE	**↑** ↑NSE<> ↑							0.47 0.58 0.56 0.45	<0.01 <0.001 <0.05 0.08	F
	↓ApSc5'	**↑**												-0.32	<0.05	L
	Chorioam		**↓**			**↓**	↓								<0.05 0.08	L
	MultPreg	**↓**													<0.005	L
						**↓**	**↓**	Late sepsis							<0.05	M
							**↑**	BPD							<0.05	M

ApSc1′, 1 min Apgar score; ApSc5′, 5 min Apgar score; BPD, bronchopulmonary dysplasia; NEC, Necrotizing enterocolitis; Chorioam, chorioamnionitis; CS, caesarean section; durHosp, duration of hospitalization; IVH, Intraventricular hemorrhage; LS, Language scale (Bayley III test); MultPreg, multiple pregnancy; ns, non-statistical significant; PreSter, prenatal steroid administration; PVL, periventricular leukomalacia; RDS, respiratory distress syndrome; RS, Receptive subscale of Language scale (Bayley III test); SBE, standard base excess.

Blue font indicates the antecedent factor in time, where red font indicates the subsequent correlated incident.

Bold font indicates statistically significant correlation.

^a^ Mean [SD].

^b^ Median [range].

^c^ Adjusted for severe RDS.

^d^ Correlation observed only in the group of prematures with encephalopathy.

^e^ Percentage of the total events seen in flow cytometry (27).

^f^ Not statistical significant after adjustment for severe RDS.

^g^ Adjusted for late sepsis.

^h^ Adjusted for NEC.

^i^ Total MRI injury score (moderate or severe injury, score > 8) (73).

* Preterms with encephalopathy (vs. preterm controls).

#### 3.2.2. Correlations

Levels of S100B in the first hours of life were correlated with the adverse event of seizures, late sepsis and death, and negatively correlated with Apgar score (1 min) and acidosis (SBE, first hours of life) (Table 2). Severe RDS (grade III/IV) was correlated with increased S100B on day 1, and thus correlations of S100B on that day with brain injury parameters were adjusted for severe RDS. Interestingly, there was trend to lower S100B levels on days 3 and 9 in neonates with mild RDS (I/II grade) compared to neonates with no RDS at all (*p* = 0.08). Caesarian section was correlated with marginally increased S100B levels on day 9. No correlation was observed between S100B levels on day 1 with gestational age, birth weight, gender, chorioamnionitis, premature rupture of membranes or prenatal steroid administration.

### 3.3. NSE

#### 3.3.1. Kinetics

NSE levels were higher in preterms with brain damage than in healthy ones at almost all time-points, but statistical significance was reached only on day 45. The subgroup of prematures that developed severe IVH (grade III/IV) had higher levels of NSE on days 3, 18 and 45. Notably, preterms with IVH/grade I had similar levels with preterms without obvious brain injury. Additionally, preterm controls had borderline higher NSE levels on days 1 and 9 compared to full-term controls. Both healthy neonate groups presented gradually lower levels over time, in contrast to preterms with encephalopathy that had elevated levels on days 18 and 45. Kinetics of NSE levels in the first 45 days of life are presented in Figure 2 and Table 2.

#### 3.3.2. Correlations

NSE levels on day 9 were borderline higher in cases of severe RDS compared to no RDS, and even more to mild RDS (*p* = 0.07). No correlation was found between NSE levels on the 1st day of life and gestational age, birth weight, gender, type of delivery, chorioamnionitis, premature rupture of membranes or prenatal steroid administration.

### 3.4. Erythropoietin

#### 3.4.1. Kinetics

EPO levels in preterms with encephalopathy were higher on days 1 to 9 compared to preterm controls, an increase attributed mainly to PVL for day 1 and mainly to severe IVH (grade III/IV) for days 3 and 9. Additionally, healthy preterms had higher EPO levels than full-term neonates on day 3, while on day 9 the opposite correlation was observed. Levels of EPO are presented in Table 2 and Figure 2.

#### 3.4.2. Correlations

**S100B** levels on days 3 and 9 were positively correlated with EPO levels of the same or the following days. Similarly, NSE levels on day 3 were positively correlated with EPO of the same day. Adversely, when seeking how EPO levels correlate with brain injury magnitude of the following days, the opposite correlation was found: EPO on day 1 was adverse correlated with S100B of the following days (Table 2).

Additionally, EPO levels were correlated with **CPCs** levels of the same or the following days, an observation found only in the group of prematures with encephalopathy (Table 2).

Severe RDS was correlated with increased EPO levels of days 1 and 3 compared to cases of mild RDS, and thus correlations of EPO on those days with brain injury were adjusted for that parameter.

Between other clinical factors, multiple pregnancy was correlated with lower EPO levels on day 1. Stress markers at birth (namely, Apgar Score (1 and 5 min) and lower pH (first hours of life)) were correlated with higher EPO on days 1 and 3. Finally, prenatal corticosteroids were borderline correlated with higher EPO levels on day 1 (adjusted for brain injury event) (Table 2).

### 3.5. Insulin-like growth factor-1

#### 3.5.1. Kinetics

Preterms with brain injury presented similar levels of IGF-1 as preterm controls, but subanalysis showed a borderline decrease in IGF-1 levels on day 18 in cases of severe IVH. Full-term neonates and healthy preterms presented gradually higher levels over time (Friedman test, *p* < 0.05), whereas preterms with encephalopathy did not. Full-term neonates had significantly higher IGF-1 levels than healthy preterms from day 9 to day 45. Levels of IGF-1 in all groups are shown in Figure 2.

#### 3.5.2. Correlations

Higher **S100B** levels (d1) and **NSE** levels (d1-d18) were correlated with lower IGF-1 levels on days 18 and 45 (Table 2). IGF-1 generally was not correlated with **CPCs** levels in any group.

Among other parameters, Apgar score (1st min) <4 was correlated with lower IGF-1 levels on days 18 and 45. Late sepsis was correlated with lower IGF-1 levels on days 9, 18 and 45. Severe RDS was correlated with lower IGF-1 levels on day 18. Lower IGF-1 levels on days 18 and 45 was correlated with the outcome of BPD as well.

Finally, low IGF-1 levels (d18, d45) were correlated with the duration of hospitalization, and low levels on day 3 and 18 with the outcome of death (Table 2). No correlation was found between IGF-1 levels of the 1st day of life and gestational age or birth weight.

### 3.6. Stromal cell-derived factor-1 (SDF-1)

#### 3.6.1. Kinetics

In the present study, SDF-1 levels were similar in all groups (Figure 2). Only the subgroup of prematures with severe IVH (III/IV grade) presented lower SDF-1 levels on day 3 and 45. Likewise, prematures with PVL had borderline lower levels on day 1 (Table 2).

#### 3.6.2. Correlations

**S100B** and **NSE** levels on day 1 were adverse correlated with SDF-1 levels on day 9. On the contrary, levels of brain injury markers of day 9 and 18 were positively correlated with SDF-1 levels of the same or the following days (Table 2). Regarding **CPCs**, SDF-1 levels on day 3 were correlated with VSELs on the same day, as were SDF-1 levels (d9, d18) with HSCs and eEPCs of day 18. Among other clinical factors, prenatal corticosteroids and chorioamnionitis were marginally correlated with lower SDF-1 on day 1. Severe RDS was correlated with increased SDF-1 levels on day 1, while SDF-1 levels on day 45 were borderline lower in cases of BPD (Table 2). No correlation was found for SDF-1 levels on day 1 and birth weight or gestational age.

### 3.7. Haematopoietic stem cells

#### 3.7.1. Kinetics

**Figure 3:**
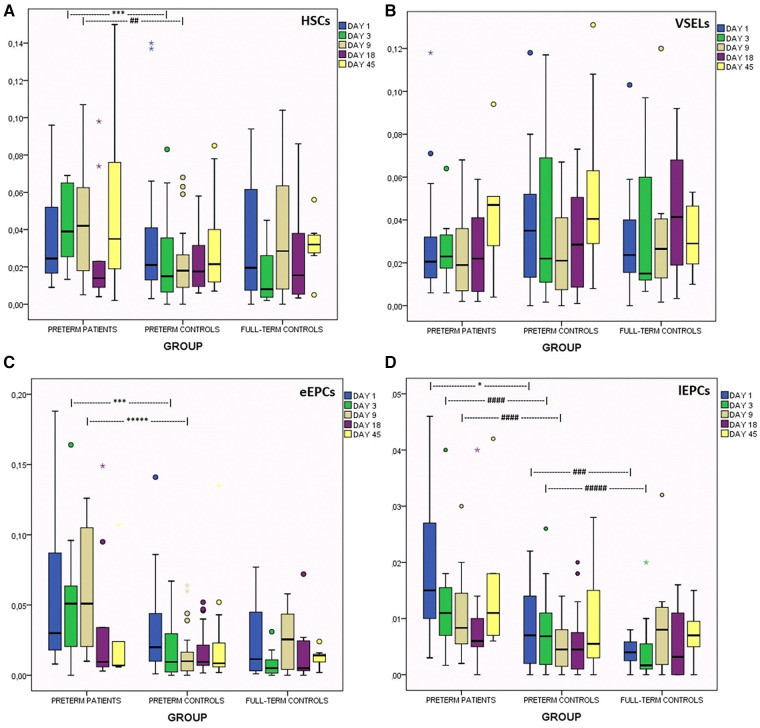
Kinetics of circulating progenitor cells in the first 45 days of life in preterms with encephalopathy, in preterm controls and in full-term controls. (**A**) HCS, (**B**) VSELs, (**C**) early EPCs, (**D**) late EPCs. *p < 0.05, ***p < 0.005, *****p < 0.0005. ^##^*p* = 0.068, ^###^*p* = 0.08, ^####^*p* = 0.056, ^####^*p* = 0.074.

In prematures with encephalopathy levels of HSCs were significantly elevated on days 3 and 9 compared to their age-controls (adjusted for NEC and late sepsis) (Figure 3, Table 2).

#### 3.7.2. Correlations

Higher **S100B** levels on day 1 were correlated with lower HSCs levels on days 3 and 9 (Table 2).

**NSE** levels on days 3 and 18 were correlated with higher HSCs levels on day 18. Among prenatal factors, chorioamnionitis was correlated with lower HSCs on days 1, 9 and 18, as well as multiple pregnancy was correlated with lower levels on days 1 and 3. Low Apgar score was correlated with higher HSCs on days 3 and 9. Interesting, low HSCs from birth to the 45th day of life were constantly associated with late sepsis, while levels on day 1 also proved to have also good prognostic ability. Additionally, low HSCs on day 3 were correlated with necrotising enterocolitis (NEC) (Table 2). Gestational age and birth weight were not correlated with HSCs (d1).

### 3.8. Very small embryonic-like stem cells

#### 3.8.1. Kinetics

VSELs' levels were found to be similar in all groups (Figure 3). The subgroup of neonates with PVL presented borderline higher VSELs on day 9 (Table 2).

#### 3.8.2. Correlations

**S100B** levels on day 1 were adverse correlated with VSELs on days 1, 3 and 45 (adjusted for late sepsis and severe RDS), whereas NSE levels on days 3 and 18 were positively correlated with VSELs of day 18 (Table 2).

Apgar score (1st min) <4 was correlated with lower VSELs on days 1, 18 and 45. A tendency for higher levels of VSELs on day 3 was found when 1st min Apgar score was between 4 and 6, compared to Apgar score higher than 6 (*p* = 0.08). VSELs levels on day 18 were higher in mild RDS, lower in neonates with no RDS at all, and much lower in severe RDS. On the same day (18th), VSELs levels were lower in cases of BPD as well. In cases of late sepsis, VSELs levels were severely depressed the whole one and a half month of life, whereas levels on day 1 presented good prognostic ability (Table 2). Finally, VSELs were not correlated with birth weight or gestational age.

### 3.9. Early endothelial progenitor cells

#### 3.9.1. Kinetics

Regarding eEPCs, prematures with brain injury had elevated levels compared to their age controls on days 3 and 9 of life (Figure 3). Subanalysis has shown that this elevation was mainly observed in neonates with severe IVH (III/IV grade), while neonates with PVL only had marginally increased levels on day 18 (Table 2).

#### 3.9.2. Correlations

NSE levels on days 3 and 18 were correlated with increased eEPCs of the same days. Multiple gestation was correlated with lower eEPCs levels on days 1, 3 and 9. Low Apgar score (1 and 5 min) was correlated with significantly higher eEPCs levels on days 3 and 9. Duration of hospitalization and occurrence of death was positively correlated with eEPCs. In cases of late sepsis, eEPCs levels were very low on days 1, 3 and 18. Levels on day 3 were also lower in cases of NEC (presenting moderate prognostic ability) (Table 2). eEPCs were not correlated with gestational age or birth weight.

### 3.10. Late endothelial progenitor cells

#### 3.10.1. Kinetics

lEPCs levels were increased in the brain injury group on days of life 1, 3 and 9, compared to the healthy preterm group. Additionally, the latter group had borderline higher levels than full-term newborns on days 1 and 3 (Figure 3).

#### 3.10.2. Correlations

**S100B** on day 1 was adversely correlated with lEPCs levels of the following days (3 and 18), whereas **NSE** was constantly positively correlated with lEPCs of the same or the following days. Low 5 min Apgar score was associated with increased lEPCs levels on day 1. Multiple gestation was correlated with lower lEPCs levels on day 1, where chorioamnionitis with lower levels on days 3, 18 and 45. Late sepsis was correlated with lower lEPCs levels on days 18 and 45, and BPD higher levels on day 45 (Table 2). lEPCs were not correlated with gestational age or birth weight.

### 3.11. Long-term neurodevelopmental outcome

25 preterms were assessed between 24 and 30 months of corrected age with Bayley III test. The neurodevelopmental outcome is shown in Table 3. Prematures with brain damage presented lower scores mostly on motor scale and expressive language subscale. S100B and NSE levels on the first day of life could prognosticate with high sensitivity and specificity retardation in the language domain (Table 2).

**Table 3:** Bayley scores in preterms with encephalopathy and in preterm controls.

Groups	Preterm Patients (Group 1)	Preterm Controls (Group 2)	Comparisons Groups (*p*)
*n*	5	20	
Cognitive scale	88 (16)	99.5 (12.1)	
Language scale	88.8 (20.7)	101.8 (16.3)	
Receptive	6.8 (4.2)	10.1 (3.3)	1–2 (0.073)
Expressive	8.8 (4.2)	10.4 (2.9)	
Motor scale	82.4 (16.4)	99 (13.6)	1–2 (<0.05)
Gross	8 (3.7)	10.8 (3.5)	1–2 (0.07)
Fine	5.6 (3.5)	8.8 (2.3)	
Cerebral palsy	1	0	

### 3.12. MRI imaging

Among preterms with encephalopathy that were alive at term-equivalent postmenstrual age, 6/7 were evaluated with MRI imaging. Two of them proved to have no significant detectable brain injury (total score <4), 1 had mild total brain injury score (5), 1 preterm had moderate score (8) and 2 of them had severe brain injury (total score ≥12). NSE levels on day 45 could prognosticate total brain injury score seen in MRI (Table 2).

Grade of white matter injury in MRI scoring was marginally correlated with lower scores in cognitive scale (*r* = −0.825/*p* = 0.08) and in motor scale (*r* = −0.392/*p* = 0.058), especially in gross motor subscale (*r* = −0.86/*p* = 0.06). Additionally, lower scores in motor scale were found in thinning of corpus callosum (gross motor subscale, *r* = −0.91, *p* < 0.05), in delayed myelination (*r* = −0.395/*p* = 0.056), in enlargement of subarachnoid space (*r* = −0.404/*p* < 0.05) and in cerebellum injury (*r* = −0.36/*p* = 0.08).

## 4. Discussion

Identifying new biomarkers which can help the clinician to make early proper therapeutic decisions is of major importance. It is desirable that these biomarkers have specific properties: they must allow accurate, instant and early detection of brain injury, they must be low-cost and minimally invasive, and finally they must be able to be applied repeatedly for longitudinal follow-up (8). Detecting and implementing certain protocols for biomarkers in clinical use may allow personalized delineation of underlining pathophysiology and instant therapeutic approaching.

### 4.1. S100B

An increase of S100B in prematures with encephalopathy was observed on the first day of life compared to preterms without detectable brain damage, a constant observation in previous studies (74–78). In accordance with our study, in most previous studies S100B levels in preterm controls were stable over time, whereas levels in preterms with encephalopathy were decreasing over time (74, 76, 78, 79). On the contrary, there were reports of cases of severe asphyxia (79) or PVL where S100B levels presented an increasing trend over time (74, 78). S100B has a very small half-life of 30–100 min (8) and thus protracted increased levels depict protracted cell necrosis or apoptosis. Additionally, we found that a cut-off point of 2.51 μg/L on the first day of life was capable of early discrimination of preterms at risk for severe IVH, whereas Metallinou et al. report a much higher cut-off point of 17.74 μg/L (75). In the current study, preterms without encephalopathy also had higher levels of brain injury biomarkers compared to full-term controls on days 1 and 9 of life. Other researchers have observed increased levels of S100B in healthy full-term neonates compared to adults, and have interpreted this phenomenon as a result of the immature brain-blood barrier in neonates (80) or the increased metabolism of neuroglial cells in this age group (81). Our finding could have a similar age-based explanation, but the possible neurotoxic implications of the relatively increased oxidative stress in preterms (although without obvious brain damage) cannot be ignored.

Regarding increased S100B levels in severe RDS, it is known that severe hypoxia could have a neurotoxic effect (9). Additionally, a ventilated premature with severe RDS could have significant fluctuations in cerebral blood flow (with the latter being a critical issue leading to IVH) (82). Moreover, in the current study S100B levels were observed to be higher in neonates with no RDS compared to ones with mild RDS (I/II grade), indicating the existence of a possible preconditioning model (mediated by mild hypercapnia and hypoxia-inducible factor-1a (HIF-1a) and its target genes, a well described model of endogenous response in the literature (3)). Similarly, after caesarian section the increased S100B levels we found could be attributed to the need of an emergency delivery (in cases of fetal distress) if the increase was observed on days 1 and 3. But having in mind the small half-life of S100B, lower S100B levels on day 9 after vaginal birth might indicate the existence of an endogenous neuroprotective preconditioning model of “mild stress” during vaginal delivery, as it has been described (83).

Moreover, the influence of preceding infection/inflammation on hypoxic-ischemic brain injury is known to be critical in preterms (4), and prenatal inflammation is known to be associated with increased maternal and fetal S100B levels (84). In the current study, we did not find correlation between prenatal inflammation and serum S100B levels in the infant, possibly due to the fact that the half-life of the marker is indeed small and the time of the blood sampling on day 1 (12–24 h of life). Additionally, there were limited cases of chorioamnionitis in the preterm patient group to establish a certain result. Also, correlation of S100B with Apgar score and acidosis was expected. Similarly, correlation of S100B on day 1 with seizures probably reflects the magnitude of brain damage and indeed the correlation of both with severe IVH. Finally, correlation with the adverse outcome of death possibly reflects the severe life-threatening medical condition. On the other hand, correlation of early S100B on day1 with late sepsis is intriguing. As S100B did not correlate with antenatal inflammation in this study (to be noted, the latter is known to correlate with postnatal inflammation (85–87), it is possible that brain injury itself affects the immune response of the newborn, becoming more vulnerable to infections.

### 4.2. NSE

We found that preterms with encephalopathy had increased levels of NSE in almost all time points, mainly attributed to the subgroup of neonates with severe IVH. A more gradual decrease of NSE over time is seen virtually in all groups, which is a reflection of the longer half-time (T_1/2_) of NSE (about 24 h) (88). But in preterms with brain injury a secondary increase is observed on days 18 and 45, indicating either enhanced prolonged apoptosis or sustained (necrotic) neural damage that is protracted for several weeks (secondary or tertiary phase). These findings are in accordance with the related pathophysiology and reflect the ongoing nature of preterm encephalopathy (3). A third presumption could be that this secondary increase is the effect of multiple comorbidities of a critical ill preterm neonate in the brain metabolism. Additionally, Giuseppe et al. reported increased NSE levels on days 1, 2 and 7 of life in preterms with severe asphyxia, compared to mild asphyxia and controls (79). Finally, as observed for S100B, preterm controls had increased NSE levels compared to healthy full-term neonates, and correlation of NSE levels with RDS was again revealed (i.e., increased NSE levels in severe RDS and a preconditioning model for mild RDS).

### 4.3. EPO

Prematures with severe IVH presented higher levels of EPO on days 3 and 9, while ones with PVL higher EPO levels on day 1 (adjusted for severe RDS). In the current study EPO was correlated with Apgar score, acidosis and RDS. It is well known that EPO is released in cases of hypoxia (via HIF-1a) (66), but constant and prolonged (till the 18th day) correlation with brain injury markers (adjusted for RDS) indicates that EPO is possibly increased also as a neurotrophic factor (independently of the presence of severe hypoxia/RDS), in the frame of an endogenous regeneration effort after CNS injury, as has been also shown in the past (24, 89). Interestingly, the higher the EPO levels on day 1, the lower the S100B levels in the following days (3 and 9), indicating once more the neuroprotective role of EPO itself, a well-established role in the literature (7, 11).This observation is also in accordance to Li et al., who found decreased serum S100B levels after EPO treatment (90).

Moreover, serum EPO levels were not associated with CPCs in preterm controls (in accordance with Bui et al. (91), but they were only elevated in the group of preterms with encephalopathy, indicating its neuroprotective action and its role as a chemoattractant for CPCs. Specifically, EPO was correlated with higher levels of HSCs (reflecting its hematopoietic role) and even more with EPCs. The latter is also observed in adults (92, 93), and it is possibly related to the release of EPO in the penumbra around the necrotic/injured cell zone and the chemotaxis of EPCs in an attempt for neoangiogenesis.

### 4.4. IGF-1

IGF-1 is a known trophic factor with additional neuroprotective and antiapoptotic properties (94, 95). It is shown to be protective to pre-OLs and promotes oligodendroglial proliferation (94). The degree of brain injury (S100B and NSE) was constantly correlated with lower IGF-1 levels in the following days, in accordance with previous observations (96–98). This means that both injury in neurons and in their microenvironment (astrocytes) lead to lower levels of a trophic and neuroprotective factor, impeding regeneration of brain injury itself and especially mediating in the pathogenesis of PVL (inability of pre-OLs' maturation). Additionally, IGF-1 is a general trophic factor (99), and decreased IGF-1 is related to inhibition of recovery of any other organ damaged in a premature neonate with multiple comorbidities in the neonatal intensive care unit (NICU). It is not surprising that, low IGF-1 was correlated with duration of hospitalization and death. A general pattern is illustrated in Figure 2: a rapid gradual increase of IGF-1 was observed mainly in healthy full-term neonates over time during the first 45 days of life, in a much lesser degree an IGF increase in preterms without encephalopathy, whereas preterm with brain injury had just measurable IGF-1 levels only in day 45 [in accordance to Hansen-Pupp et al. (100)]. Inability of preterms patients to increase IGF-1 levels over time might mirror the most severe clinical condition of these neonates in the NICU that inhibits the release of a trophic factor. Similarly, IGF-1 increase was inhibited by other stress or inflammation conditions as well: very low Apgar score, severe RDS and late sepsis. IGF-1 was not proved to be a chemoattractant for the cell lines of interest in the current study. IGF-1 is known to be gradually increasing during pregnancy (101), which explains its lower levels observed in prematures compared to full-term neonates in the current study.

### 4.5. SDF-1

Although studies in adults constantly showed increased serum SDF-1 levels after tissue injury (57, 102), studies in neonates are not so conclusive (103–105). In adults, the main role of SDF-1 is chemotaxis in cases of tissue injury (57, 102, 106). In utero, SDF-1 is implicated in mobilization of progenitor cells for organogenesis (54), a procedure that is abruptly interrupted after premature delivery. It is possible that premature transition from intrauterine to extrauterine life implicates signals from tissue injury, especially in the first days of life. Initial adverse correlation of S100B and NSE levels on day 1 with latter SDF-1 levels might depict the abrupt inhibition of normal SDF-1 activity *in utero* after premature birth. In our study, brain injury was correlated positively with SDF-1 [i.e., presenting a role of chemotaxis after injury, as seen in adult models (57, 102)] in full-term neonates (data not shown) and in preterms only after day 9 of life. More studies are needed for a conclusive theory (103, 104, 107). Additionally, in this study, SDF-1 levels were increased in full-term newborns compared to preterms, but this has not reached statistical significance, in accordance to other researchers (103, 104). Machalinska et al. (107) have found increased levels in full-term neonates at the 10th week of life. We also observed positive (but not constant) correlations of SDF-1 levels with CPCs, that are indicative of its role as a chemoattractant. It is certain, that in neonates more factors are implicated, especially in a neonate in the setting of critical illness and therefore more studies are required.

### 4.6. CPCs

Correlations between brain injury markers and CPCs are of great interest. S100B was constantly related to low CPCs' levels of the same or the following days, whereas NSE was correlated to increased CPCs levels over time. As this is the first study to our knowledge that CPCs are studied together with brain injury markers, we can only make assumptions about this observation. In an ideal model, in cases that only neuronal injury is taking place (i.e., NSE increasement), the intact micro-environment of the neurons (i.e., astrocytes and endothelium) will release chemoattractants in an effort to recruit neuroprotective factors, as CPCs from the periphery. But when cytotoxic damage includes the micro-environment as well (i.e., astrocyte injury, with S100B increasement), signals via chemoattractants cannot be released and CPCs are not mobilized from periphery towards the brain. Another explanation might issue at the same time. As it is known that neurons are more vulnerable than astrocytes to hypoxia (7), NSE is a more sensitive brain injury marker and it is increased in an milder hypoxic event, where only neurons are damaged and astrocytes are spared. When S100B is increased there is an indication of a more severe brain damage and a more severe general clinical condition, in which both chemotaxis chain and peripheral mobilization of CPCs are affected. It seems that in more severe clinical conditions (hypoxic or oxidative), neither the “signal” is released, nor the recipient (CPCs) is capable to respond.

Another general observation in the current study is the inhibited mobilization of many cell lines in cases of inflammation, both antenatally (chorioamnionitis) and postnatally (late sepsis and NEC). It is well known that prenatal inflammation can predispose to postnatal inflammation (108–110), the latter being the second main mechanism (together with ischemia) that lead to preterm white matter injury (3, 7). If reduction of CPCs is the mediated step between antenatal and postnatal inflammation (i.e., antenatal inflammation leads to decreased CPCs, and decreased CPCs predispose to postnatal infection), or reduced CPCs are the 3rd hit in the pathway to white matter injury [taking into account their trophic actions in cases organogenesis and in cases of tissue injury as well (35–37)] this needs to be clarified in the future. Moreover, we observed that especially late sepsis was constantly correlated with decreased CPCs levels at almost any time point during the first one and a half month of life. At the end of the day, inflammation of any origin results in reduced CPCs, i.e., reduced regenerative ability for tissue injuries of any origin.

It is essential to be aware that different studies in the literature characterize cell lines in a different way, and this is a major problem for comparisons and for certain conclusions to be drawn. The less antibodies are used to identify cell lines, the less specific is the cell line characterization. E.g., CD34^+^ cells are found in HSCs but also in VSELs and EPCs. Below reports of different studies will be presented, but absolute comparisons are not possible.

#### 4.6.1. HSCs

In the current study, HSCs were found to be elevated in preterms with encephalopathy on days 3 and 9, but no differences were found on day 1. That means that HSCs’ levels probably do not play a preventive role for the future appearance of brain damage, but rather HSCs are elevated secondarily as a response to the occurrence of brain injury.

In agreement with the present study, Borghesi et al. found no correlation of CD34^+^/CD45^+^ cells of day 1 with IVH, but correlation on day 3 with PVL (111). Likewise, Paviotti et al. did not find correlation on day 1 for CD34^+^ cells (112). On the contrary, Kotowski et al. report decreased levels of HSCs (as CD184^+^/lin^−^/CD45^+^) in the umbilical cord in newborns that had later IVH or any complication of prematurity, indicating a possible protective role of these cells (104).

We found no correlation of HSCs with gestational age and birth weight, as Kotowski et al. (measuring CD184^+^lin^−^CD45^+^ cells) (104) and Paviotti et al.(CD34^+^) (112) did, but most researchers that measure CD34^+^ (91, 105, 113–115) or (CD34^+^CD45^+^) (111) cells did find an inverse correlation with gestational age.

#### 4.6.2. VSELs

In the current study VSELs kinetics was similar in all groups, with a trend to gradually increase from day 9 to day 45. Only subgroup of preterms with IVH had higher levels on day 9. Kotowski et al. also report steady levels in the first 6 weeks of life (CD184^+^lin^−^CD45^−^) and a trend to higher levels in prematures compared to full-term babies (104). Machalinska et al. report increased levels in preterms at the 10th week of life (107). We found no correlation of levels on day 1 with birth weight, but Kotowski et al. reported higher levels in low birth weight prematures. Regarding correlation with Apgar score and RDS, the common preconditioning pattern was observed once again: higher VSELs levels in mild stress (Apgar score between 4 and 6) compared to VSELs levels when Apgar score was >6, and much lower VSELs levels in severe stress (Apgar score <4). Similarly, higher VSELS levels were observed in mild RDS compared to no RDS at all (where severe RDS presents the lower VSELS levels). It seems that in mild stress the endogenous regeneration mechanisms are mobilized, but in severe stress these mechanisms are assaulted and are unable to help endogenous regeneration. Identifying neonates with suppressed endogenous mechanisms might be a target for exogenous administration of neuroprotective substances or progenitor cells in the future.

#### 4.6.3. eEPCs

EPCs are novel markers of angiogenesis, playing a vital role in vascular growth and repair (59). eEPCs were found increased in preterms with encephalopathy (mainly with IVH), on days 3 and 9 of life. Strauss et al. found elevated endothelial RNA markers after the 2nd week of life (116), where Safranow et al. found elevated EPCs in cord blood in those neonates (103). eEPCs were also elevated in cases of low Apgar score in the current study, as also in Safranow et al. The above observation indicates that increasement of EPCs levels is probably a secondary process as a response to stress/brain injury, whereas increased levels in cord blood that were reported by Safranow et al. need to be further studied. Other researchers could not establish a correlation of eEPCs with brain damage (111, 112). Correlation with duration of hospitalization and death probably depicts the severity of the clinical condition, in which the eEPCs are elevated. We found no correlation of eEPCs on day 1 with birth weight and gestational age, whereas Safranow et al. found inverse correlation (103).

#### 4.6.4. lEPCs

lEPCs were found increased in preterms with encephalopathy and in low Apgar score [in accordance with Safranow et al. (103)], indicating that perinatal stress and brain injury mobilize pluripotent progenitor cells with neoangiogenetic and regeneration abilities. Elevated lEPCs in healthy preterms compared to healthy full-term neonates depicts the ongoing process of organogenesis and angiogenesis in the fetus, disrupted suddenly by the preterm birth [in accordance with Safranow et al. (103)]. Similarly, increased lEPCs on day 45 in neonates with BPD might depict a trend for enhanced neoangiogenesis.

The main limitation of the current study is the relatively small number of patients, especially after the second week of life (due to the adverse event of death), that did not allow multiple regression analysis for confounding factors. This was a single-center study, with long follow up, and thus a relatively small number of patients could be enrolled. Nevertheless, absence of correlation between biomarkers and several important perinatal characteristics (as gestational age, birth weight, type of delivery or premature rupture of membranes) decreases importantly the number of possible confounders. Additionally, when confounders were observed (e.g., sepsis, RDS, NEC), confirmation of observed correlations after exclusion of neonates with these parameters did establish the results. Moreover, some observations were reported having only borderline significance when there was a meaningful rationale or when constant patterns were observed, because the scope of this pilot study was to highlight unknown pathophysiological pathways and correlations, that would be clarified by future studies, and not to come to final conclusions.

In conclusion, in this study we aimed to investigate if various preclinical observations are valid in preterm neonates, measuring multiple parameters in a single study and in multiple time-points. S100B and NSE proved to be very sensitive markers of brain injury, illustrating the time and extent of brain injury (hypothesis A). Additionally, they proved to have good prognostic ability for neuroimaging (S100B could prognosticate severe IVH, and NSE MRI scoring—hypothesis G) and language deficits at 2 years of corrected age (hypothesis H). These biomarkers are well studied, relatively low-cost and minimally invasive, and it seems that their clinical utility would be of great value. Regarding chemoattractants and neurotrophic factors, EPO was intensively increased in preterm brain injury, whereas IGF-1 and SDF-1 were rather decreased (hypothesis B). Similarly, CPCs were intense mobilized in those neonates, mainly HSCs and EPCs (hypothesis C) (Figure 1, Table 2). EPO and SDF-1 proved to act as chemoattractants for CPCs, but IGF-1 did not (hypothesis D). EPO also was observed to act as a neuroprotective factor itself, while IGF-1 indicated its trophic role as well (hypothesis E). Mobilization of CPCs was intense and not fully attributed to the chemoattractants we studied, indicating that other factors mediated as well (hypothesis F). Additionally, antenatal factors did not correlate with brain injury markers (hypothesis I), but multiple pregnancy, prenatal steroid administration and chorioamnionitis had moderate impact on EPO and SDF-1 levels (hypothesis K) and more intense impact on levels of CPCs (hypothesis L). Finally, correlation of chemoattractants and CPCs with comorbidities (namely RDS, sepsis and NEC) was rather impressive (hypothesis M), delineating relation of inflammation with brain injury and related outcome. It is of special interest that a repeated preconditioning model was observed, with clinical importance that could be very valuable for future therapeutical strategies.

Increased chemoattractants and CPCs after brain injury, as well as decreased trophic factors and inability of CPCs mobilization in severe clinical conditions, delineate the endogenous regeneration effort or its suppression respectively. Individualized identification of the critical ill neonate whose endogenous regeneration mechanisms are suppressed could be a target for exogenous administration of neurotrophic factors or progenitor/stem cells in the near future.

## Data Availability

The raw data supporting the conclusions of this article will be made available by the authors, without undue reservation.

## References

[1] Cao G Liu J Liu M. Global, regional, and national incidence and mortality of neonatal preterm birth, 1990–2019. JAMA Pediatr. (2022) 176:787–96. 10.1001/jamapediatrics.2022.1622 35639401 PMC9157382

[2] Stoll BJ Hansen NI Bell EF Walsh MC Carlo WA Shankaran S Trends in care practices, morbidity, and mortality of extremely preterm neonates, 1993–2012. JAMA. (2015) 314:1039–51. 10.1001/jama.2015.10244 26348753 PMC4787615

[3] Stevenson DK Benitz WE Sunshine P Hintz SR Druzin ML, editors. Fetal and neonatal brain injury. 4th ed. New York: Cambridge University Press (2009). 10.1017/CBO9780511581281

[4] Lagercrantz H Hanson MA Ment LR Peebles DM, editors. The newborn brain. 2nd ed. New York: Cambridge University Press (2010). 10.1017/CBO9780511711848

[5] Folkerth RD Kinney HC. Perinatal neuropathology. In: Louis DN Love S Ellison DW, editors. Greenfield's neuropathology, 8th ed. London: Arnold (2009). Vol. 1, p. 402.

[6] Volpe JJ. Commentary—do the negative results of the PENUT trial close the book on erythropoietin for premature infant brain? J Neonatal Perinatal Med. (2020) 13:149–52. 10.3233/NPM-200444 32333558 PMC7369037

[7] Volpe JJ Inder TE Darras BT de Vries LS du Plessis AJ Neil J Volpe’s neurology of the newborn. 6th ed. Philadelphia: Elsevier (2018). 1240.

[8] Efstathiou N. Biomarkers in neonatal brain injury: interpreting research into clinical practice. In: Rajendram R Preedy VR Patel VB, editors. Biomarkers in trauma, injury and critical care. Biomarkers in disease: Methods, discoveries and applications. Cham: Springer (2022). p. 549–96. 10.1007/978-3-030-87302-8_72

[9] Swaiman K Ashwal S Ferriero D Schor N, editors. Swaiman’s pediatric neurology. 5th ed. Elsevier (2011). ISBN: 978-1437704358.

[10] Efstathiou N Slavakis A Drossou V Kantziou K Dermetzoglou V Soubasi V. Can we delineate brain injury in full-term neonates using serum biomarkers? Brain Inj. (2021) 35:821–30. 10.1080/02699052.2021.1907862 33780304

[11] Jantzie L El Demerdash N Newville JC Robinson S. Time to reconsider extended erythropoietin treatment for infantile traumatic brain injury? Exp Neurol. (2019) 318:205–15. 10.1016/j.expneurol.2019.05.004 31082389

[12] Rangarajan V Juul SE. Erythropoietin: emerging role of erythropoietin in neonatal neuroprotection. Pediatr Neurol. (2014) 51:481–8. 10.1016/j.pediatrneurol.2014.06.008 25266611 PMC4180944

[13] Spandou E Papoutsopoulou S Soubasi V Karkavelas G Simeonidou C Kremenopoulos G Hypoxia-ischemia affects erythropoietin and erythropoietin receptor expression pattern in the neonatal rat brain. Brain Res. (2004) 1021:167–72. 10.1016/j.brainres.2004.06.057 15342264

[14] Mu D Chang YS Vexler ZS Ferriero DM. Hypoxia-inducible factor 1alpha and erythropoietin upregulation with deferoxamine salvage after neonatal stroke. Exp Neurol. (2005) 195:407–15. 10.1016/j.expneurol.2005.06.001 16023639

[15] Castillo-Meléndez M Yan E Walker DW. Expression of erythropoietin and its receptor in the brain of late-gestation fetal sheep, and responses to asphyxia caused by umbilical cord occlusion. Dev Neurosci. (2005) 27:220–7. 10.1159/000085995 16046857

[16] Iwai M Cao G Yin W Stetler RA Liu J Chen J. Erythropoietin promotes neuronal replacement through revascularization and neurogenesis after neonatal hypoxia/ischemia in rats. Stroke. (2007) 38:2795–803. 10.1161/STROKEAHA.107.483008 17702962

[17] Ohls RK Cannon DC Phillips J Caprihan A Patel S Winter S Preschool assessment of preterm infants treated with darbepoetin and erythropoietin. Pediatrics. (2016) 137:e20153859. 10.1542/peds.2015-3859 26908704 PMC4771132

[18] Spandou E Papadopoulou Z Soubasi V Karkavelas G Simeonidou C Pazaiti A Erythropoietin prevents long-term sensorimotor deficits and brain injury following neonatal hypoxia-ischemia in rats. Brain Res. (2005) 1045:22–30. 10.1016/j.brainres.2005.03.013 15910759

[19] Song J Sun H Xu F Kang W Gao L Guo J Recombinant human erythropoietin improves neurological outcomes in very preterm infants. Ann Neurol. (2016) 80:24–34. 10.1002/ana.24677 27130143 PMC5084793

[20] Natalucci G Latal B Koller B Rüegger C Sick B Held L Effect of early prophylactic high-dose recombinant human erythropoietin in very preterm infants on neurodevelopmental outcome at 2 years: a randomized clinical trial. JAMA. (2016) 315:2079–85. 10.1001/jama.2016.5504 27187300

[21] Juul SE Comstock BA Wadhawan R Mayock DE Courtney SE Robinson T A randomized trial of erythropoietin for neuroprotection in preterm infants. N Engl J Med. (2020) 382:233–43. 10.1056/nejmoa1907423 31940698 PMC7060076

[22] Bierer R Peceny MC Hartenberger CH Ohls RK. Erythropoietin concentrations and neurodevelopmental outcome in preterm infants. Pediatrics. (2006) 118:e635–640. 10.1542/peds.2005-3186 16908620

[23] He J-S Huang Z-L Yang H Weng K-Z Zhu S-B. Early use of recombinant human erythropoietin promotes neurobehavioral development in preterm infants. Zhongguo Dang Dai Er Ke Za Zhi. (2008) 10:586–8. PMID: . 18947475

[24] Juul SE Stallings SA Christensen RD. Erythropoietin in the cerebrospinal fluid of neonates who sustained CNS injury. Pediatr Res. (1999) 46(5):543–7. 10.1203/00006450-199911000-00009 10541316

[25] Wei X Du Z Zhao L Feng D Wei G He Y IFATS Collection: the conditioned media of adipose stromal cells protect against hypoxia-ischemia-induced brain damage in neonatal rats. Stem Cells. (2009) 27:478–88. 10.1634/stemcells.2008-0333 19023032

[26] Lapidot T Petit I. Current understanding of stem cell mobilization: the roles of chemokines, proteolytic enzymes, adhesion molecules, cytokines, and stromal cells. Exp Hematol. (2002) 30:973–81. 10.1016/S0301-472X(02)00883-4 12225788

[27] Efstathiou N Soubasi V Koliakos G Kyriazis G Zafeiriou DI Slavakis A Mobilization of circulating progenitor cells following brain injury in premature neonates could be indicative of an endogenous repair process. A pilot study. Hippokratia. (2015) 19:141–7. PMID: . 27418763 PMC4938105

[28] van Velthoven CTJ Kavelaars A van Bel F Heijnen CJ. Mesenchymal stem cell treatment after neonatal hypoxic-ischemic brain injury improves behavioral outcome and induces neuronal and oligodendrocyte regeneration. Brain Behav Immun. (2010) 24:387–93. 10.1016/j.bbi.2009.10.017 19883750

[29] Park KI Hack M Ourednik J Yandava B Flax JD Stieg PE Acute injury directs the migration, proliferation, and differentiation of solid organ stem cells: evidence from the effect of hypoxia-ischemia in the CNS on clonal “reporter” neural stem cells. Exp Neurol. (2006) 199:156–78. 10.1016/j.expneurol.2006.04.002 16737696

[30] Miller JT Bartley JH Wimborne HJC Walker AL Hess DC Hill WD The neuroblast and angioblast chemotaxic factor SDF-1 (CXCL12) expression is briefly up regulated by reactive astrocytes in brain following neonatal hypoxic-ischemic injury. BMC Neurosci. (2005) 6:63. 10.1186/1471-2202-6-63 16259636 PMC1298306

[31] Hill WD Hess DC Martin-Studdard A Carothers JJ Zheng J Hale D SDF-1 (CXCL12) is upregulated in the ischemic penumbra following stroke: association with bone marrow cell homing to injury. J Neuropathol Exp Neurol. (2004) 63:84–96. 10.1093/jnen/63.1.84 14748564

[32] Wang Y Deng Y Zhou G-Q. SDF-1alpha/CXCR4-mediated migration of systemically transplanted bone marrow stromal cells towards ischemic brain lesion in a rat model. Brain Res. (2008) 1195:104–12. 10.1016/j.brainres.2007.11.068 18206136

[33] Aicher A Zeiher AM Dimmeler S. Mobilizing endothelial progenitor cells. Hypertension. (2005) 45:321–5. 10.1161/01.HYP.0000154789.28695.ea 15655116

[34] Aiuti A Webb IJ Bleul C Springer T Gutierrez-Ramos JC. The chemokine SDF-1 is a chemoattractant for human CD34+ hematopoietic progenitor cells and provides a new mechanism to explain the mobilization of CD34+ progenitors to peripheral blood. J Exp Med. (1997) 185:111–20. 10.1084/jem.185.1.111 8996247 PMC2196104

[35] Carroll JE Borlongan C V. Adult stem cell therapy for acute brain injury in children. CNS Neurol Disord Drug Targets. (2008) 7:361–9. 10.2174/187152708786441812 18991664

[36] Borlongan C V Hadman M Sanberg CD Sanberg PR. Central nervous system entry of peripherally injected umbilical cord blood cells is not required for neuroprotection in stroke. Stroke. (2004) 35:2385–9. 10.1161/01.STR.0000141680.49960.d7 15345799

[37] Wang M Crisostomo PR Herring C Meldrum KK Meldrum DR. Human progenitor cells from bone marrow or adipose tissue produce VEGF, HGF, and IGF-I in response to TNF by a p38 MAPK-dependent mechanism. Am J Physiol Regul Integr Comp Physiol. (2006) 291:R880–884. 10.1152/ajpregu.00280.2006 16728464

[38] Vannucci RC Vannucci SJ. A model of perinatal hypoxic-ischemic brain damage. Ann N Y Acad Sci. (1997) 835:234–49. 10.1111/j.1749-6632.1997.tb48634.x 9616778

[39] Iwai M Ikeda T Hayashi T Sato K Nagata T Nagano I Temporal profile of neural stem cell proliferation in the subventricular zone after ischemia/hypoxia in the neonatal rat brain. Neurol Res. (2006) 28:461–8. 10.1179/016164105X49283 16759450

[40] Muñoz-Elías G Woodbury D Black IB. Marrow stromal cells, mitosis, and neuronal differentiation: stem cell and precursor functions. Stem Cells. (2003) 21:437–48. 10.1634/stemcells.21-4-437 12832697

[41] Hara K Yasuhara T Maki M Matsukawa N Masuda T Yu SJ Neural progenitor NT2N cell lines from teratocarcinoma for transplantation therapy in stroke. Prog Neurobiol. (2008) 85:318–34. 10.1016/j.pneurobio.2008.04.005 18514379

[42] Hess DC Borlongan CV. Stem cells and neurological diseases. Cell Prolif. (2008) 41(Suppl 1):94–114. 10.1111/j.1365-2184.2008.00486.x 18181951 PMC6496373

[43] Lin KK Goodell MA. Detection of hematopoietic stem cells by flow cytometry. Methods Cell Biol. (2011) 103:21–30. 10.1016/B978-0-12-385493-3.00002-4 21722798

[44] Verina T Fatemi A Johnston MV Comi AM. Pluripotent possibilities: human umbilical cord blood cell treatment after neonatal brain injury. Pediatr Neurol. (2013) 48:346–54. 10.1016/j.pediatrneurol.2012.10.010 23583051

[45] Lapidot T Dar A Kollet O. How do stem cells find their way home? Blood. (2005) 106:1901–10. 10.1182/blood-2005-04-1417 15890683

[46] Nervi B Link DC DiPersio JF. Cytokines and hematopoietic stem cell mobilization. J Cell Biochem. (2006) 99:690–705. 10.1002/jcb.21043 16888804

[47] Papayannopoulou T Scadden DT. Stem-cell ecology and stem cells in motion. Blood. (2008) 111:3923–30. 10.1182/blood-2007-08-078147 18398055 PMC2288715

[48] Dar A Kollet O Lapidot T. Mutual, reciprocal SDF-1/CXCR4 interactions between hematopoietic and bone marrow stromal cells regulate human stem cell migration and development in NOD/SCID chimeric mice. Exp Hematol. (2006) 34:967–75. 10.1016/j.exphem.2006.04.002 16863903

[49] Taguchi A Soma T Tanaka H Kanda T Nishimura H Yoshikawa H Administration of CD34+ cells after stroke enhances neurogenesis via angiogenesis in a mouse model. J Clin Invest. (2004) 114:330–8. 10.1172/JCI200420622 15286799 PMC484977

[50] Hennemann B Ickenstein G Sauerbruch S Luecke K Haas S Horn M Mobilization of CD34+ hematopoietic cells, colony-forming cells and long-term culture-initiating cells into the peripheral blood of patients with an acute cerebral ischemic insult. Cytotherapy. (2008) 10:303–11. 10.1080/14653240801949994 18418775

[51] Taguchi A Matsuyama T Moriwaki H Hayashi T Hayashida K Nagatsuka K Circulating CD34-positive cells provide an index of cerebrovascular function. Circulation. (2004) 109:2972–5. 10.1161/01.CIR.0000133311.25587.DE 15184275

[52] Dunac A Frelin C Popolo-Blondeau M Chatel M Mahagne MH Philip PJM. Neurological and functional recovery in human stroke are associated with peripheral blood CD34+ cell mobilization. J Neurol. (2007) 254:327–32. 10.1007/s00415-006-0362-1 17345048

[53] Ratajczak MZ Kim CH Wojakowski W Janowska-Wieczorek A Kucia M Ratajczak J. Innate immunity as orchestrator of stem cell mobilization. Leukemia. (2010) 24:1667–75. 10.1038/leu.2010.162 20703253

[54] Ratajczak MZ Zuba-Surma E Kucia M Reca R Wojakowski W Ratajczak J. The pleiotropic effects of the SDF-1-CXCR4 axis in organogenesis, regeneration and tumorigenesis. Leukemia. (2006) 20:1915–24. 10.1038/sj.leu.2404357 16900209

[55] Ratajczak M Zuba-Surma E. Very small embryonic-like stem cells: characterization, developmental origin, and biological significance. Experimental. (2008) 36:742–51. 10.1016/j.exphem.2008.03.010 PMC2430762 18474305

[56] Kucia M Zhang YP Reca R Wysoczynski M Machalinski B Majka M Cells enriched in markers of neural tissue-committed stem cells reside in the bone marrow and are mobilized into the peripheral blood following stroke. Leukemia. (2006) 20:18–28. 10.1038/sj.leu.2404011 16270036

[57] Paczkowska E Kucia M Koziarska D Halasa M Safranow K Masiuk M Clinical evidence that very small embryonic-like stem cells are mobilized into peripheral blood in patients after stroke. Stroke. (2009) 40:1237–44. 10.1161/STROKEAHA.108.535062 19246697

[58] Yoder MC. Human endothelial progenitor cells. Cold Spring Harb Perspect Med. (2012) 2:a006692. 10.1101/cshperspect.a006692 22762017 PMC3385946

[59] Asahara T Kawamoto A Masuda H. Concise review: circulating endothelial progenitor cells for vascular medicine. Stem Cells. (2011) 29:1650–5. 10.1002/stem.745 21948649

[60] Hunting CB Noort WA Zwaginga JJ. Circulating endothelial (progenitor) cells reflect the state of the endothelium: vascular injury, repair and neovascularization. Vox Sang. (2005) 88:1–9. 10.1111/j.1423-0410.2005.00589.x 15663716

[61] Zhang ZG Zhang L Jiang Q Chopp M. Bone marrow-derived endothelial progenitor cells participate in cerebral neovascularization after focal cerebral ischemia in the adult mouse. Circ Res. (2002) 90:284–8. 10.1161/hh0302.104460 11861416

[62] Fadini GP. An underlying principle for the study of circulating progenitor cells in diabetes and its complications. Diabetologia. (2008) 51:1091–4. 10.1007/s00125-008-1021-0 18478199

[63] Fadini GP Losordo D Dimmeler S. Critical reevaluation of endothelial progenitor cell phenotypes for therapeutic and diagnostic use. Circ Res. (2012) 110:624–37. 10.1161/CIRCRESAHA.111.243386 22343557 PMC3382070

[64] Chen JZ Zhang FR Tao QM Wang XX Zhu JH Zhu JH. Number and activity of endothelial progenitor cells from peripheral blood in patients with hypercholesterolaemia. Clin Sci (Lond). (2004) 107:273–80. 10.1042/CS20030389 15099190

[65] Umemura T Soga J Hidaka T Takemoto H Nakamura S Jitsuiki D Aging and hypertension are independent risk factors for reduced number of circulating endothelial progenitor cells. Am J Hypertens. (2008) 21:1203–9. 10.1038/ajh.2008.278 18787520

[66] Gonzalez FF Ferriero DM. Neuroprotection in the newborn infant. Clin Perinatol. (2009) 36:859–80. 10.1016/j.clp.2009.07.013 19944839 PMC2786822

[67] Gazzolo D Marinoni E di Iorio R Lituania M Bruschettini PL Michetti F. Circulating S100beta protein is increased in intrauterine growth-retarded fetuses. Pediatr Res. (2002) 51:215–9. 10.1203/00006450-200202000-00015 11809917

[68] Loukovaara M Teramo K Alfthan H Hämäläinen E Stefanovic V Andersson S. Amniotic fluid S100B protein and erythropoietin in pregnancies at risk for fetal hypoxia. Eur J Obstet Gynecol Reprod Biol. (2009) 142:115–8. 10.1016/j.ejogrb.2008.10.008 19042077

[69] Florio P Marinoni E Di Iorio R Bashir M Ciotti S Sacchi R Urinary S100B protein concentrations are increased in intrauterine growth-retarded newborns. Pediatrics. (2006) 118:e747–754. 10.1542/peds.2005-2875 16923924

[70] Netto CBO Portela L V Ferreira CT Kieling C Matte U Felix T Ontogenetic changes in serum S100B in down syndrome patients. Clin Biochem. (2005) 38:433–5. 10.1016/j.clinbiochem.2004.12.014 15820773

[71] Albers CA Grieve AJ. Test review: bayley, N. (2006). bayley scales of infant and toddler development—third edition. San antonio, TX: harcourt assessment. J Psychoeduc Assess. (2007) 25:180–90. 10.1177/0734282906297199

[72] Velikos K Soubasi V Michalettou I Sarafidis K Nakas C Papadopoulou V Bayley-III scales at 12 months of corrected age in preterm infants: patterns of developmental performance and correlations to environmental and biological influences. Res Dev Disabil. (2015) 45–46:110–9. 10.1016/j.ridd.2015.07.014 26232203

[73] Kidokoro H Neil JJ Inder TE. New MR imaging assessment tool to define brain abnormalities in very preterm infants at term. Am J Neuroradiol. (2013) 34:2208–14. 10.3174/ajnr.A3521 23620070 PMC4163698

[74] Chiang LM Chen WY Yang YC Jeng MJ. Elevation of serum S100 protein concentration as a marker of ischemic brain damage in extremely preterm infants. J Chinese Med Assoc. (2015) 78:610–6. 10.1016/j.jcma.2015.06.009 26285828

[75] Metallinou D Karampas G Nyktari G Iacovidou N Lykeridou K Rizos D. S100b as a biomarker of brain injury in premature neonates. A prospective case—control longitudinal study. Clin Chim Acta. (2020) 510:781–6. 10.1016/j.cca.2020.09.013 32941837

[76] Zhou W Li W Qu LH Tang J Chen S Rong X. Relationship of plasma S100B and MBP with brain damage in preterm infants. Int J Clin Exp Med. (2015) 8:16445–53. PMID: . 26629170 PMC4659058

[77] Distefano G Curreri R Betta P Isaja MT Romeo MG Amato M. Serial protein S-100 serum levels in preterm babies with perinatal asphyxia and periventricular white matter lesions. Am J Perinatol. (2002) 19:317–22. 10.1055/s-2002-34463 12357423

[78] Huang RZ Zhang YJ Zhang JF Su YM Peng LQ Ya N. Relation between prognosis and changes of MBP and S100B in premature infants with periventricular leukomalacia. Genet Mol Res. (2015) 14:4338–43. 10.4238/2015.April.30.6 25966206

[79] Giuseppe D Sergio C Pasqua B Giovanni LV Salvatore C Frigiola A Perinatal asphyxia in preterm neonates leads to serum changes in protein S-100 and neuron specific enolase. Curr Neurovasc Res. (2009) 6:110–6. 10.2174/156720209788185614 19442160

[80] Maschmann J E Heinemann MK Ziemer G Speer CP. Evaluation of protein S-100 serum concentrations in healthy newborns and seven newborns with perinatal acidosis. Acta Paediatr. (2000) 89:553–5. 10.1111/j.1651-2227.2000.tb00337.x 10852191

[81] Portela LVC Tort ABL Schaf D V Ribeiro L Nora DB Walz R The serum S100B concentration is age dependent. Clin Chem. (2002) 48:950–2. 10.1093/clinchem/48.6.950 12029017

[82] Perlman JM McMenamin JB Volpe JJ. Fluctuating cerebral blood-flow velocity in respiratory-distress syndrome. Relation to the development of intraventricular hemorrhage. N Engl J Med. (1983) 309:204–9. 10.1056/NEJM198307283090402 6866033

[83] Gidday JM. Cerebral preconditioning and ischaemic tolerance. Nat Rev Neurosci. (2006) 7:437–48. 10.1038/nrn1927 16715053

[84] Garnier Y Frigiola A Li Volti G Florio P Frulio R Berger R Increased maternal/fetal blood S100B levels following systemic endotoxin administration and periventricular white matter injury in preterm fetal sheep. Reprod Sci. (2009) 16:758–66. 10.1177/1933719109335801 19525402

[85] Chau V Brant R Poskitt KJ Tam EWY Synnes A Miller SP. Postnatal infection is associated with widespread abnormalities of brain development in premature newborns. Pediatr Res. (2012) 71:274–9. 10.1038/pr.2011.40 22278180 PMC3940469

[86] Shah DK Doyle LW Anderson PJ Bear M Daley AJ Hunt RW Adverse neurodevelopment in preterm infants with postnatal sepsis or necrotizing enterocolitis is mediated by white matter abnormalities on magnetic resonance imaging at term. J Pediatr. (2008) 153:170–5.e1. 10.1016/j.jpeds.2008.02.033 18534228

[87] Stoll BJ Hansen NI Adams-Chapman I Fanaroff AA Hintz SR Vohr B National institute of child health and human development neonatal research network. Neurodevelopmental and growth impairment among extremely low-birth-weight infants with neonatal infection. JAMA. (2004) 292:2357–65. 10.1001/jama.292.19.2357 15547163

[88] Roka A Kelen D Halasz J Beko G Azzopardi D Szabo M. Serum S100B and neuron-specific enolase levels in normothermic and hypothermic infants after perinatal asphyxia. Acta Paediatr Int J Paediatr. (2012) 101:319–23. 10.1111/j.1651-2227.2011.02480.x 21981269

[89] Juul SE McPherson RJ Bauer LA Ledbetter KJ Gleason CA Mayock DE. A phase I/II trial of high-dose erythropoietin in extremely low birth weight infants: pharmacokinetics and safety. Pediatrics. (2008) 122(2):383–91. 10.1542/peds.2007-2711 18676557

[90] Li Z-M Xiao Y-L Zhu J-X Geng F-Y Guo C-J Chong Z-L Recombinant human erythropoietin improves functional recovery in patients with severe traumatic brain injury: a randomized, double blind and controlled clinical trial. Clin Neurol Neurosurg. (2016) 150:80–3. 10.1016/j.clineuro.2016.09.001 27611985

[91] Bui KCT Weems M Biniwale M George AA Zielinska E Azen CG Circulating hematopoietic and endothelial progenitor cells in newborn infants: effects of gestational age, postnatal age and clinical stress in the first 3 weeks of life. Early Hum Dev. (2013) 89:411–8. 10.1016/j.earlhumdev.2012.12.006 23312395 PMC3633695

[92] Bahlmann FH De Groot K Spandau J-M Landry AL Hertel B Duckert T Erythropoietin regulates endothelial progenitor cells. Blood. (2004) 103:921–6. 10.1182/blood-2003-04-1284 14525788

[93] Yip H-K Tsai T-H Lin H-S Chen S-F Sun C-K Leu S Effect of erythropoietin on level of circulating endothelial progenitor cells and outcome in patients after acute ischemic stroke. Crit Care. (2011) 15:R40. 10.1186/cc10002 21269484 PMC3221969

[94] Lin S Fan LW Pang Y Rhodes PG Mitchell HJ Cai Z. IGF-1 protects oligodendrocyte progenitor cells and improves neurological functions following cerebral hypoxia-ischemia in the neonatal rat. Brain Res. (2005) 1063:15–26. 10.1016/j.brainres.2005.09.042 16259966

[95] Hansen-Pupp I Hövel H Löfqvist C Hellström-Westas L Fellman V Hüppi PS Circulatory insulin-like growth factor-I and brain volumes in relation to neurodevelopmental outcome in very preterm infants. Pediatr Res. (2013) 74:564–9. 10.1038/pr.2013.135 23942554

[96] Wan Z-H Xiao X. Relationship between serum levels of insulin-like growth factor I and growth hormone and neonatal hypoxic-ischemic encephalopathy. Zhongguo Dang Dai Er Ke Za Zhi. (2007) 9:22%–24. PMID: . 17306071

[97] Satar M Ozcan K Yapicioğlu H Narli N. Serum insulin-like growth factor 1 and growth hormone levels of hypoxic-ischemic newborns. Biol Neonate. (2004) 85:15–20. 10.1159/000074952 14631161

[98] Dinleyici EC Tekin N Colak O Aksit MA. Cord blood IGF-1 and IGFBP-3 levels in asphyxiated term newborns. Neuroendocrinol Lett. (2006) 27:745–7. PMID: . 17187018

[99] https://en.wikipedia.org/wiki/Insulin-like_growth_factor_1.

[100] Hansen-Pupp I Löfqvist C Polberger S Niklasson A Fellman V Hellström A Influence of insulin-like growth factor i and nutrition during phases of postnatal growth in very preterm infants. Pediatr Res. (2011) 69:448–53. 10.1203/PDR.0b013e3182115000 21263374

[101] Bennett A Wilson DM Liu F Nagashima R Rosenfeld RG Hintz RL. Levels of insulin-like growth factors I and II in human cord blood. J Clin Endocrinol Metab. (1983) 57:609–12. 10.1210/jcem-57-3-609 6348065

[102] Wojakowski W Tendera M Kucia M Zuba-Surma E Paczkowska E Ciosek J Mobilization of bone marrow-derived oct-4+ SSEA-4+ very small embryonic-like stem cells in patients with acute myocardial infarction. J Am Coll Cardiol. (2009) 53:1–9. 10.1016/j.jacc.2008.09.029 19118716 PMC5536894

[103] Safranow K Kotowski M Lewandowska J Machalińska A Dziedziejko V Czajka R Circulating endothelial progenitor cells in premature infants: is there an association with premature birth complications? J Perinat Med. (2012) 40:455–62. 10.1515/jpm-2011-0199 22752779

[104] Kotowski MJ Safranow K Kawa MPMP Lewandowska J Kłos P Dziedziejko V Circulating hematopoietic stem cell count is a valuable predictor of prematurity complications in preterm newborns. BMC Pediatr. (2012) 12:148. 10.1186/1471-2431-12-148 22985188 PMC3573966

[105] Qi Y Qian L Sun B Chen C Cao Y. Circulating CD34+ cells are elevated in neonates with respiratory distress syndrome. Inflamm Res. (2010) 59(10):889–95. 10.1007/s00011-010-0201-9 20431906

[106] Drukała J Paczkowska E Kucia M Młyńska E Krajewski A Machaliński B Stem cells, including a population of very small embryonic-like stem cells, are mobilized into peripheral blood in patients after skin burn injury. Stem Cell Rev. (2012) 8:184–94. 10.1007/s12015-011-9272-4 21573962

[107] Machalinska A Modrzejewska M Kotowski M Dziedziejko V Kucia M Kawa M Circulating stem cell populations in preterm infants: implications for the development of retinopathy of prematurity. Arch Ophthalmol. (2010) 128:1311–9. 10.1001/archophthalmol.2010.221 20938001

[108] Yanowitz TD Baker RW Roberts JM Brozanski BS. Low blood pressure among very-low-birth-weight infants with fetal vessel inflammation. J Perinatol. (2004) 24:299–304. 10.1038/sj.jp.7211091 15042111

[109] Yanowitz TD Potter DM Bowen A Baker RW Roberts JM. Variability in cerebral oxygen delivery is reduced in premature neonates exposed to chorioamnionitis. Pediatr Res. (2006) 59:299–304. 10.1203/01.pdr.0000196738.03171.f1 16439596 PMC4074908

[110] Yanowitz TD Ann Jordan J Gilmour CH Towbin R Bowen A Roberts JM Hemodynamic disturbances in premature infants born after chorioamnionitis: association with cord blood cytokine concentrations. Pediatr Res. (2002) 51:310–6. 10.1203/00006450-200203000-00008 11861935

[111] Borghesi A Massa M Campanelli R Bollani L Tzialla C Figar TA Circulating endothelial progenitor cells in preterm infants with bronchopulmonary dysplasia. Am J Respir Crit Care Med. (2009) 180:540–6. 10.1164/rccm.200812-1949OC 19574444

[112] Paviotti G Fadini GP Boscaro E Agostini C Avogaro A Chiandetti L Endothelial progenitor cells, bronchopulmonary dysplasia and other short-term outcomes of extremely preterm birth. Early Hum Dev. (2011) 87:461–5. 10.1016/j.earlhumdev.2011.03.011 21511414

[113] Bizzarro MJ Bhandari V Krause DS Smith BR Gross I. Circulating stem cells in extremely preterm neonates. Acta Paediatr. (2007) 96:521–5. 10.1111/j.1651-2227.2007.00194.x 17391470

[114] Li K Yau FW Fok TF So KW Li CK Yuen PM. Haematopoietic stem and progenitor cells in human term and preterm neonatal blood. Vox Sang. (2001) 80:162–9. 10.1046/j.1423-0410.2001.00025.x 11449956

[115] Opie TM Shields LE Andrews RG. Cell-surface antigen expression in early and term gestation fetal hematopoietic progenitor cells. Stem Cells. (1998) 16:343–8. 10.1002/stem.160343 9766814

[116] Strauss T Metsuyanim S Pessach I Shuchan-Eisen I Kuint J Dekel B. Analysis of circulating hem-endothelial marker RNA levels in preterm infants. BMC Pediatr. (2009) 9:42. 10.1186/1471-2431-9-42 19555479 PMC2709108

